# Human papillomavirus vaccine delivery practices among pediatricians and pediatric trainees in a tertiary hospital in Singapore

**DOI:** 10.1002/pdi3.102

**Published:** 2024-07-26

**Authors:** Grace Yan Ling Ler, Sudipta Roy Chowdhury

**Affiliations:** ^1^ Department of General Pediatrics and Adolescent Medicine Kandang Kerbau Women's and Children's Hospital Singapore Singapore; ^2^ Duke-National University of Singapore Medical School Singapore Singapore; ^3^ Lee Kong Chian School of Medicine Nanyang Technological University Singapore Singapore; ^4^ Yong Loo Lin School of Medicine National University of Singapore Singapore Singapore

**Keywords:** HPV‐related cancers, human papillomavirus infection, human papillomavirus vaccine, immunization, pediatricians, vaccine advocacy, vaccine barriers, vaccine knowledge

## Abstract

Human Papillomavirus (HPV) is the most common sexually transmitted infection and is associated with cervical, anogenital, and oropharyngeal cancers. It is crucial to improve vaccination uptake in both genders as primary prevention for these conditions. Pediatricians play an active role in advocating for HPV vaccination and our study aims to assess the level of knowledge, attitudes, barriers, and practices among pediatric trainees and general pediatricians regarding HPV infection and vaccination. A survey‐questionnaire was administered to our target groups. It comprised 14 questions regarding demographics of the healthcare provider, knowledge of HPV infection and vaccination, practices and barriers of recommending HPV vaccination, and effective strategies for improving HPV vaccine uptake. Among survey respondents, majority did not recommend for HPV vaccination (66.7%) or receive any enquiry about it (80.6%) within the preceding 12 months. The most common perceived barrier was inadequate knowledge, which was consistent with the misconceptions regarding HPV infection and vaccination that were identified in this survey. Strategies which physicians felt would be most effective in increasing vaccine uptake include educating and providing resources for both physicians and caregivers as well as making the vaccine free. Our study revealed a low advocacy rate for HPV vaccination. Physicians need to be equipped with the knowledge, skills, and resources to better counsel caregivers as well as focus our efforts on vaccinating male patients in order to increase vaccine uptake in both genders.

## Graphical abstract

This study showed the human papillomavirus (HPV) vaccination advocacy trends, perceived barriers, and facilitators for pediatricians to initiate HPV vaccination regime. By identifying the barriers for vaccination, we aim to work toward improving vaccine uptake rates, especially among the young male population to improve herd immunity.

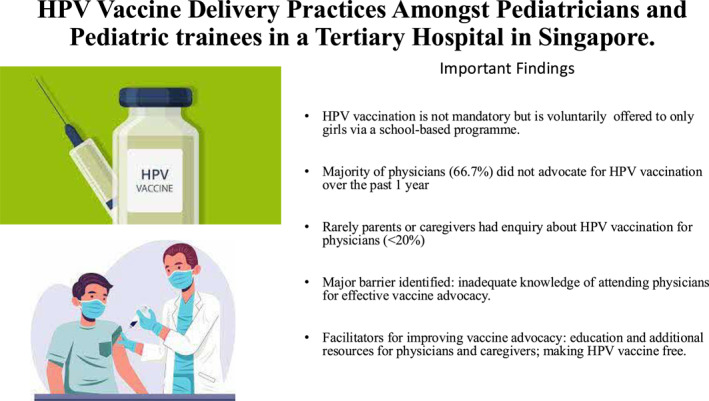

## 1 INTRODUCTION

Cervical cancer is the fourth most common cancer in women worldwide with an estimated 604,000 new cases and 342,000 deaths in 2020.^1^ In Singapore, cervical cancer ranks as the 11th leading cause of female cancer for all ages and the 5th most common cancer in women aged 15–44 years. It also ranks as the 8th leading cause of cancer deaths among women, with 172 deaths from it annually.^2^

Human papillomavirus (HPV) is the most common sexually transmitted infection (STI), with 90% of cervical cancer cases caused by 9 high‐risk strains of HPV.^3^ The strong association between HPV and cervical cancer has been well established, with the carcinogenic strains HPV 16 and 18 contributing to over 70% of cases worldwide.^4^ Moreover, HPV infections are also associated with anogenital and oropharyngeal cancers. Current preventive strategies for cervical cancer include primary prevention with HPV vaccination as well as secondary prevention with regular screening with Papanicolaou smear or HPV assays.

Children in Singapore receive immunizations based on the National Childhood Immunization Schedule (NCIS) through primary healthcare services or regular school health visits, which are conducted by the Health Promotion Board. Vaccinations are on an opt‐in basis except for vaccinations against Measles and Diphtheria, which are mandated by the Infectious Diseases Act in Singapore. The HPV vaccination was first introduced into NCIS in 2010 and is recommended for females aged 9–26 years old in Singapore. It has been cost‐effective and a good strategy to reduce the impact of HPV infection.^5^ There are currently two HPV vaccines approved for use in Singapore― bivalent Cervarix vaccine and nonavalent Gardasil 9 vaccine. Young females are entitled to free HPV vaccination through the school‐based program or by receiving a subsidy under the NCIS if they are Singapore citizens or permanent residents. Although males are not included in the recommendation, they may still choose to get vaccinated^6^ but are not eligible for any subsidy. Prior to the introduction of the school‐based vaccination program, which offers HPV vaccination to Secondary 1 (13 years old) female students in Singapore on an opt‐in basis, only 13.6% of women aged 18–26 years were immunized against HPV.^7^ Although HPV vaccination rates have increased after the introduction of this program, more can be done to further increase the uptake and to encourage students to complete the 2‐dose vaccination course. Furthermore, we need to encourage HPV vaccination in young male patients as the rate of HPV‐related cancers in this population is increasing in prevalence.^8^

Pediatrician recommendations are often considered the most effective method in encouraging HPV vaccination initiation in children.^9^ We can play a crucial role in advocating for the safety and efficacy of the HPV vaccine and assisting parents to make informed decisions. However, there is limited literature on knowledge, attitudes, and practices regarding HPV infection and vaccination in the Singapore context, especially among pediatricians.

The purpose of this study is to assess the level of knowledge, attitudes, barriers, and practices among pediatric trainees and general pediatricians regarding HPV infection and vaccination. We also examined effective implementation strategies that can improve our HPV vaccination uptake. This is the first known paper discussing about pediatricians' knowledge, perceived barriers, and delivery practices for HPV vaccination in Singapore.

## 2 MATERIALS AND METHODS

### 2.1 Survey administration

A survey‐questionnaire was created using an online secure survey platform, which would keep the respondents anonymous. All pediatric trainees and general pediatricians in Kandang Kerbau Women's and Children's Hospital, which is a 827‐bed Joint Commission International (JCI)‐accredited tertiary hospital, were informed of this survey and the online survey link was distributed via emails and text reminders. Participation was voluntary and responses were collected over 8 weeks between March 2023 and April 2023. Up to two reminders were sent via text messages for non‐respondents.

### 2.2 Survey design

The survey comprised 14 questions (see Supporting Information S1) which aims to understand the (a) demographics of the healthcare provider, (b) knowledge of HPV infection and vaccination, (c) practices and barriers of recommending HPV vaccination, and (d) effective strategies for improving HPV vaccine uptake.

For the knowledge questions regarding HPV infection and vaccination, a correct answer was given a score of 1 point each and the total scores out of 14 were computed for each respondent to assess the overall knowledge level. Scores less than 50% were categorized as poor, 50%–80% were categorized as average, and more than 80% were categorized as good knowledge base.

### 2.3 Data analysis

Statistical analysis was performed using the SPSS 28.0 (IBM Corp.) statistical software program. Data are reported as frequency (%) when applicable. Subgroup analysis was done using Chi square tests with statistical significance level set at *p* < 0.05.

The study was exempted from our local Institutional Review Board (IRB) review as it was considered a service evaluation and categorized under a Quality Improvement (QI) project.

## 3 RESULTS

The survey response rate was 80.0% (44/55) among pediatric residents and 50.0% (28/56) among general paediatricians, with a total survey response rate of 64.9% (72/111). Most of the respondents were below 40 years of age (83.3%). Table 1 shows the demographics of the survey respondents.

**TABLE 1:** Demographics of survey respondents.

	*N* (%)
Gender	
Female	46 (63.9)
Male	26 (36.1)
Age	
26–30 years	18 (25.0)
31–40 years	42 (58.3)
41–50 years	9 (12.5)
51–60 years	3 (4.2)
Race	
Chinese	55 (76.4)
Indian	9 (12.5)
Others	7 (9.7)
Malay	1 (1.4)
Religion	
Christianity	32 (44.4)
Free‐thinker/atheist	21 (29.2)
Buddhism	12 (16.7)
Hinduism	5 (6.9)
Muslim	2 (2.8)
Current position	
Year 1 residency	8 (11.1)
Year 2 residency	9 (12.5)
Year 3 residency	5 (6.9)
Year 4 residency	7 (9.7)
Year 5 residency	6 (8.3)
Year 6 residency	5 (6.9)
Clinical associate/registrar	4 (5.6)
Associate consultant	10 (13.9)
Consultant	11 (15.3)
Senior consultant and above	7 (9.7)

### 3.1 Knowledge regarding HPV infection and vaccination

**FIGURE 1:**
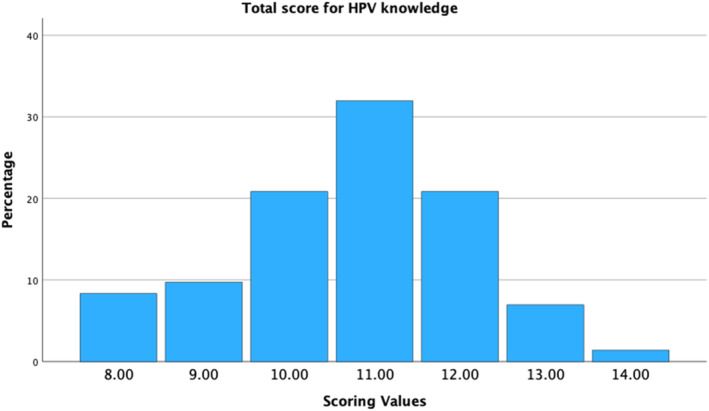
HPV knowledge assessment scores across all respondents. HPV, human papillomavirus.

Misconceptions that were identified include the ranking of cervical cancer among women in Singapore and the strains of HPV causing genital warts and cervical cancer. Regarding HPV vaccination, a significant proportion (less than 75%) of physicians were unsure if it is a part of school‐based vaccination program, the minimum age to receive it, if booster dose is required, and the number of available HPV vaccines in Singapore (Table 2). None of the respondents had poor knowledge as the lowest score was 8 out of 14 (57.1%). Majority of respondents (51/72, 70.8%) had average knowledge with a minority (21/72, 29.2%) having a good knowledge base (Figure 1). There was no statistical difference in knowledge levels (average vs. good knowledge) between pediatric trainees and pediatricians.

**TABLE 2:** Knowledge questions regarding HPV infection and vaccination answered correctly.

Knowledge prompt	Residents and registrars *N* = 44 *n* (%)	Associate consultants and above *N* = 28 *n* (%)
Cervical cancer ranks among the top 5 cancer among women in Singapore.	5 (11.4)	3 (10.7)
Genital warts are caused by the same strains of HPV that cause cervical cancer.	26 (59.1)	15 (53.6)
HPV is an uncommon sexually‐transmitted infection.	42 (95.5)	27 (96.4)
Most of HPV infections are symptomatic.	42 (95.5)	24 (85.7)
Only girls or women can be given HPV vaccination.	41 (93.2)	26 (92.9)
HPV vaccination is part of Health Promotion Board school‐based vaccination program.	18 (40.9)	21 (75)
A possible side effect of the HPV vaccine is infertility.	43 (97.7)	27 (96.4)
The minimum age limit for receiving HPV vaccination is 9 years.	22 (50)	18 (64.3)
HPV vaccines cannot be claimed by Medisave.	43 (97.7)	28 (100)
Booster dose is needed for additional protection from HPV.	29 (65.9)	16 (57.1)
HPV vaccine is only recommended prior to a patient being sexually active.	39 (88.6)	25 (89.3)
HPV vaccine is currently not available in KKH pediatric clinics and wards.	43 (97.7)	27 (96.4)
HPV vaccination only consists of a single dose.	43 (97.7)	28 (100)
There are 2 types of HPV vaccines currently available in Singapore.	31 (70.5)	21 (75)

Abbreviations: HPV, human papillomavirus; KKH, Kandang Kerbau Hospital.

### 3.2 Current practices regarding HPV vaccination

**FIGURE 2:**
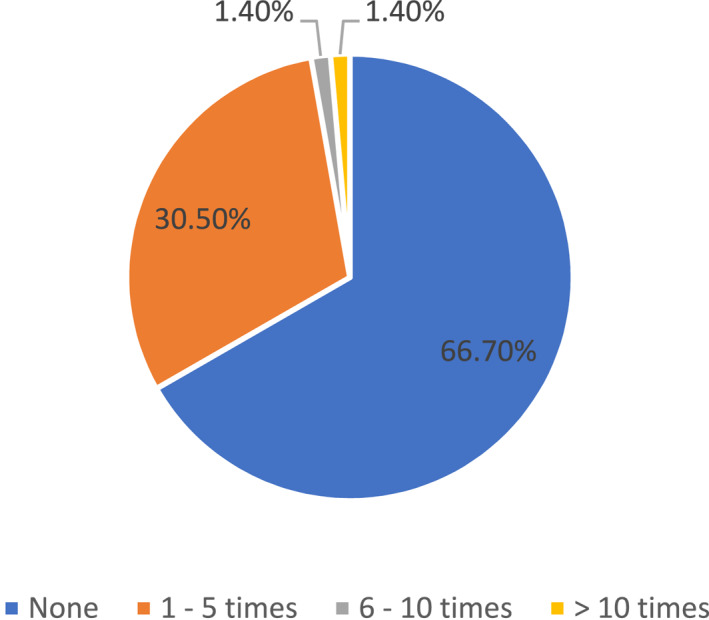
Frequency of physicians recommending HPV vaccination in past 12 months (%). HPV, human papillomavirus.

**FIGURE 3:**
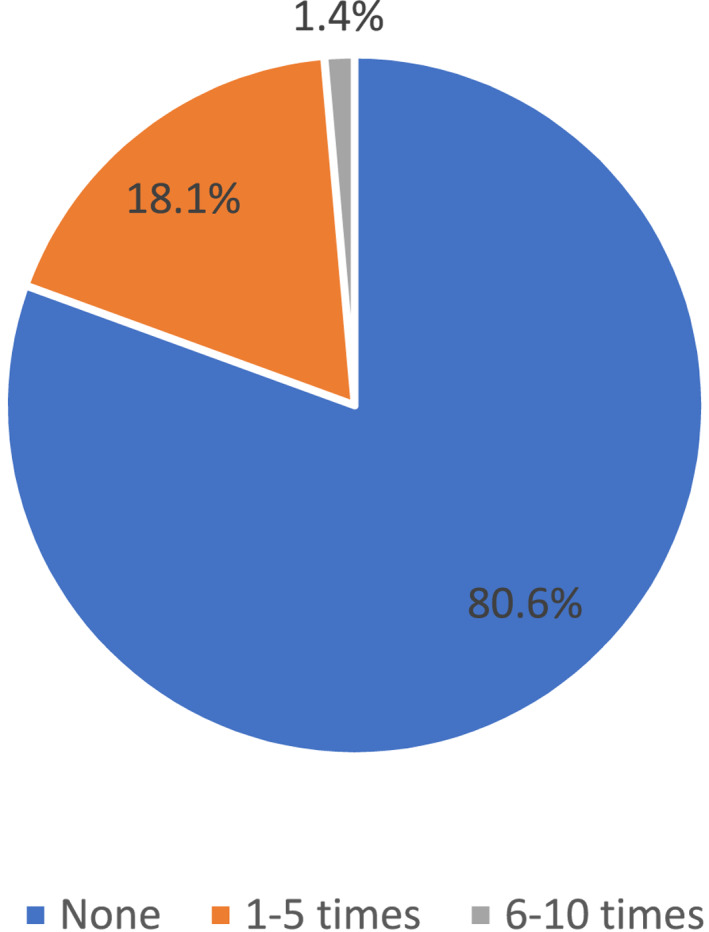
Frequency of patient or caregiver inquiring about HPV vaccination in past 12 months (%). HPV, human papillomavirus.

Within the preceding 12 months, majority of the physicians (48/72, 66.7%) did not advocate for HPV vaccination to their patients at all. A small minority had recommended the vaccine 1–5 times (22/72, 30.5%) with even fewer physicians recommending it more frequently than this (2/72, 2.8%) (Figure 2). Majority of physicians (58/72, 80.6%) did not encounter any patient or caregiver inquiring about HPV vaccination during their consultations within the preceding 12 months (Figure 3).

### 3.3 Perceived barriers to HPV vaccine delivery

**FIGURE 4:**
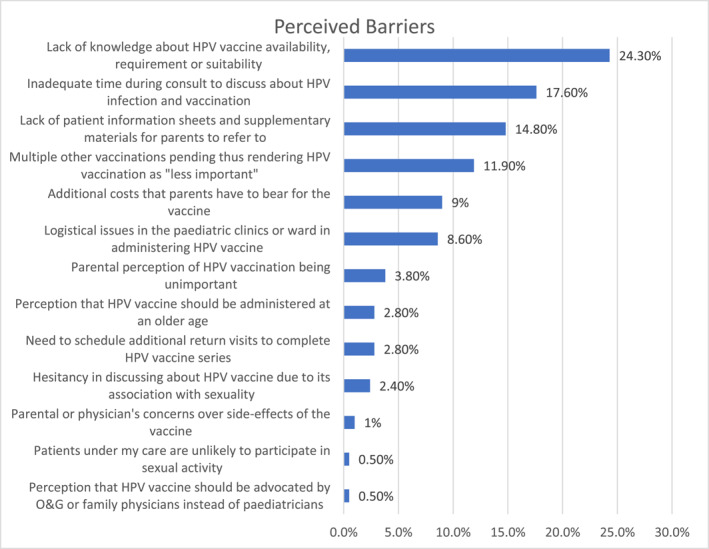
Perceived barriers to HPV vaccine delivery (%). HPV, human papillomavirus; O&G, Obstetrics and Gynecology.

There were many perceived barriers identified that contributed to the lack of physician recommendation including knowledge and cultural barriers, logistical issues, concerns regarding cost and safety profile of the vaccine. Most commonly cited reasons were inadequate knowledge about HPV vaccination (24.3%), insufficient time during consultations to discuss about HPV infection and vaccination (17.6%), as well as a lack of educational materials for patients and parents (14.8%) (Figure 4).

### 3.4 Strategies to improve HPV vaccination uptake

**FIGURE 5:**
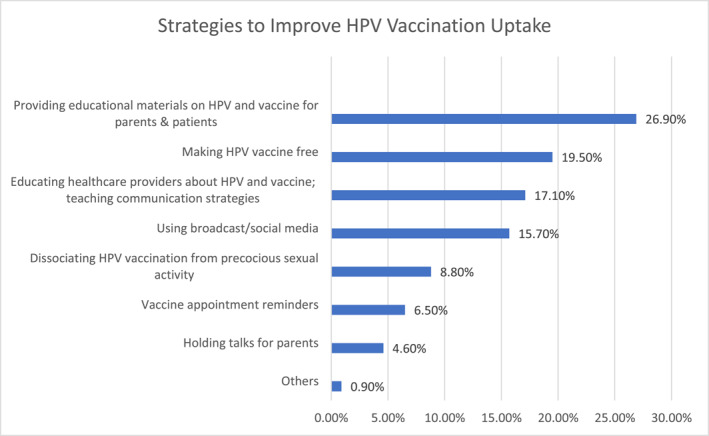
Strategies to improve HPV vaccination uptake (%). HPV, human papillomavirus.

It is important to review strategies to overcome barriers and improve vaccine uptake. Strategies that were suggested addressed the issues of (a) inadequate knowledge by providing resources to physicians to allow them to be more confident in advocating for the vaccine as well as to caregivers and patients to increase awareness of the vaccine and for them to make an informed decision, (b) cultural barriers by addressing the perception that HPV vaccine would promote early sexual initiation, (c) logistical issues such as by providing timely reminders for vaccine appointments and (d) cost concerns with a policy change of making HPV vaccine free in hospitals or clinics. The top 3 strategies which physicians felt would be most effective were (1) providing educational materials on HPV and vaccine for parents & patients (26.9%), (2) making HPV vaccination free (19.5%) and (3) educating healthcare providers about HPV and vaccine; teaching communication strategies (17.1%) (Figure 5).

## 4 DISCUSSION

In Singapore, 1 out of 10 women are affected with HPV infection and 50% of this group have high‐risk HPV strains.^10^ This is similar to worldwide trends where HPV infection is a common STI^11^ with highest prevalence in Asia and Africa. HPV is not only significant for invasive cervical cancer but has been implicated in the rise of anogenital cancers^12^ and oropharyngeal cancers worldwide.^13^ Despite the significant healthcare burden for HPV, vaccination uptake has decreased worldwide recently due to barriers imposed by the recent COVID‐19 pandemic, which has placed this vaccine at a lower vaccine priority.^14^ Hence, it is important for us to re‐focus on primary prevention to ensure optimal health outcomes for our future generations.

It has been well established that HPV vaccine uptake is higher when primary care physicians and pediatricians take an active role of recommending it through patient education and anticipatory guidance.^15^ Even in unvaccinated patients, parents would re‐consider their decision to vaccinate if their primary physician recommends for it.^16^ However, our study has shown that majority of physicians (66.7%) did not recommend for HPV vaccination even once in the preceding 12 months. This is similar to other studies where there is significant hesitancy in discussing HPV vaccination by healthcare providers.^17^ The most important reason cited was the lack of physician knowledge regarding HPV infection and vaccination in our center. This is evident as only a minority of physicians (29.2%) had good knowledge scores for the survey we conducted (Figure 1) and there were multiple misconceptions that were identified through the knowledge questions (Table 2). In addition, the lack of recommendation for HPV vaccination was also attributed to clinic time constraints and a lack of patient educational materials for HPV vaccination. These barriers are common in other centers as well.^18^ Other barriers that have been identified in previous studies include parental fears of early sexual initiation,^19^ lack of information on HPV vaccine,^20^ vaccine safety^16^ and cost of the vaccine.^15^ Moreover, due to conservative Asian values in our society, there is often apprehension in initiating conversation on the topic of HPV infection, which is related to sexual activity and STI. However, studies have shown that HPV vaccination is not related to increased sexual activity in teenagers.^21^

With the initiation of HPV vaccination, the burden of cervical cancer has been reduced.^22^ However, the prevalence of HPV‐related oropharyngeal and anorectal cancer continue to increase worldwide^23^ and in Singapore^8^ with a rising emergence of HPV‐associated conditions in men.^24^ Hence, HPV vaccination is now also routinely recommended for males in other developed countries such as Canada^25^ and the United Kingdom.^26^ It is thus prudent for our pediatricians to also recommend HPV vaccination to young male patients, who are currently not included in the NCIS, to protect them from infection. Gender‐neutral vaccination for HPV would effectively prevent HPV‐associated diseases in both genders, reduce transmission, and hopefully facilitate the eradication of HPV.

Interventions to ensure increased HPV vaccination uptake has to be through education for the parents and physicians and this was also highlighted significantly in our survey results (Figure 5). This can be done by implementing continued medical education activities with credits^27^ for healthcare providers to encourage participation, conducting resident training, and engaging pharmaceutical companies to assist with both reminder and recall systems. Another important strategy is for HPV vaccination to be free which has also been highlighted in other studies.^28^ Currently, young females are entitled to free HPV vaccination through the school‐based program or by receiving a subsidy under the NCIS, whilst males are not eligible for any subsidy for the vaccine. Hence, a policy change to grant subsidies for our male patients need to be considered to ensure that the HPV vaccination remains affordable for all.

Raising awareness of the importance of HPV vaccination and providing resources to patients and caregivers is also essential in improving its uptake. A Singapore study^29^ regarding our school‐based vaccination program revealed worrying trends. Majority of parents (56%) had never heard of HPV vaccination. Among those who did (44%), majority received their information from physicians (30%) rather than other sources such as the internet (23%) or social media (9%). Our survey also revealed that majority of physicians (80.6%) did not encounter any enquiry about the HPV vaccination in the past 12 months (Figure 3). This reinforces the importance of opportunistically educating parents, which would most likely be effective through medical professionals for trusted and accurate information. It also highlighted the lack of resources available in other mediums such as social media, which has been an important source of information for parents and children in Singapore. Hence, greater use of social media, which was highlighted as an important strategy in our survey, can be used to further increase awareness. This Singapore study also revealed that majority of parents who declined the vaccination did not specify their reasons (44.6%) or had concerns regarding its side effects (14.4%). Physicians will thus need to address the concerns of these parents and clarify misconceptions so that they can make an informed decision.

Our survey revealed dismal results of majority of physicians not actively advocating for HPV vaccination or receiving any inquiry about it in the preceding 12 months. Hence, there is an urgent need to re‐focus on our approach to advocating for HPV vaccination. In addition, many parents are completely unaware of this vaccine^29^ or may be hesitant to broach this topic as they may associate HPV vaccination with an STI. Given the changing social trends resulting in early sexual initiation worldwide, pediatricians should have a greater role in advocating for the vaccine as HPV vaccination is the most effective if received before initiation of sexual activity.^30^ It is thus our obligation and duty as pediatricians to initiate this very important conversation with parents and patients.

This is the first known study regarding knowledge, perceived barriers, and delivery practices for HPV vaccination among pediatricians in Singapore. However, limitations of this study are that it is a cross‐sectional study conducted in a single tertiary center, which affects the generalizability of results. Furthermore, the response rate among general pediatricians is low (28/56, 50.0%) although there was excellent response from the trainees (44/55, 80.0%). After implementation of the proposed strategies, further studies should be conducted to assess if they are effective in helping pediatricians to advocate for HPV vaccine and improve its uptake rate.

## 5 CONCLUSION

This study revealed the lack of recommendation for HPV vaccination by pediatricians in our center, which was largely contributed by inadequate knowledge, as well as clinic time constraints and lack of patient resources. As advocates for HPV vaccination, we need to be equipped with the knowledge, skills, and resources to better counsel parents and patients. We should also focus our efforts on vaccinating our male patients who are not included in the NCIS. Parents and physicians are the driving force to ensure improving HPV vaccination rates hence we need to engage both these target groups simultaneously to facilitate readiness for HPV vaccination during healthcare visits.

## AUTHOR CONTRIBUTIONS

All authors have read and approved the final manuscript. Sudipta Roy Chowdhury and Grace Yan Ling Ler performed the research and designed the research study. Sudipta Roy Chowdhury analyzed the data. Grace Yan Ling Ler wrote the paper. Both authors reviewed and edited the manuscript and approved the final version of the manuscript.

## CONFLICT OF INTEREST STATEMENT

The authors declared no potential conflicts of interest with respect to the research, authorship, and/or publication of this article.

## ETHICS STATEMENT

This study was exempted by the SingHealth Institutional Review Board as it was categorized under service evaluation and was considered as a QI project. Ethical approval was waived by our local Institutional Review Board (IRB) as it was considered a service evaluation and categorized under a QI project.

## INFORMED CONSENT

Informed consent was implied as the survey was voluntary and the completion and participation of the survey was optional.

## Supporting information

Supporting Information S1

## Data Availability

The data that support the findings of this study are available from the corresponding author upon reasonable request.
