# Supplementary material for: Human papillomavirus vaccine delivery practices among pediatricians and pediatric trainees in a tertiary hospital in Singapore
Source: Pediatr Discov. 2024 Jul 26;2(3):e102. doi: 10.1002/pdi3.102 (PMC12118293; doi:10.1002/pdi3.102)
Supplement: Supplementary file 1 — Supporting Information S1 [file PDI3-2-e102-s001.docx]

**Questionnaire on advocating for HPV vaccination as paediatricians**

Instructions

Please fill up this survey for us to better understand the current practices and barriers for advocating for human papilloma virus (HPV) vaccine in our paediatric population.

Questions

1. What is your age group?
   - < 21 years
   - 21 – 25 years
   - 26 – 30 years
   - 31 – 35 years
   - 36 – 40 years
   - 41 – 45 years
   - 46 – 50 years
   - 51 – 55 years
   - 56 – 60 years
   - > 60 years
2. What is your gender?
   - Male
   - Female
3. What is your race?
   - Chinese
   - Malay
   - Indian
   - Others:
4. What is your religious belief?
   - Christianity
   - Buddhism
   - Muslim
   - Hinduism
   - Free-thinker/Atheist
   - Others:
5. How many years have you worked as a doctor?
   - < 5 years
   - 5 – 10 years
   - 11 – 15 years
   - 16 – 20 years
   - > 20 years
6. What is your current position at work?
   - Year 1 Residency
   - Year 2 Residency
   - Year 3 Residency
   - Year 4 Residency
   - Year 5 Residency
   - Year 6 Residency
   - Clinical Associate/Registrar (if not in residency)
   - Associate consultant
   - Consultant
   - Senior consultant and above
7. How frequently have you recommended HPV vaccine in the past 12 months?
   - 0
   - 1 – 5 times
   - 6 – 10 times
   - > 10 times
8. How frequently has a parent or patient enquired about HPV vaccine during your consultations in the past 12 months?
   - 0
   - 1 – 5 times
   - 6 – 10 times
   - > 10 times
9. The following statements regarding HPV infection and vaccine are true: (Tick all that apply)
   - Only girls/women can be given HPV vaccination.
   - HPV vaccination is part of HPB school-based vaccination programme.
   - Cervical cancer ranks among the top 5 cancer among women in Singapore.
   - A possible side effect of the HPV vaccine is infertility.
   - The minimum age limit for receiving HPV vaccination is 9 years.
   - Genital warts are caused by the same strains of HPV that cause cervical cancer.
   - HPV is an uncommon sexually-transmitted infection.
   - HPV vaccines cannot be claimed by Medisave.
   - Most of HPV infections are symptomatic.
   - Booster dose is needed for additional protection from HPV.
   - HPV vaccine is only recommended prior to a patient being sexually active.
   - HPV vaccine is currently not available in KKH paediatric clinics and wards.
   - HPV vaccination only consists of a single dose.
   - There are 2 types of HPV vaccines currently available in Singapore.
10. What makes it challenging for you to advocate for HPV vaccine? (Tick all that apply)
    - Lack of knowledge about HPV vaccine availability, requirement or suitability.
    - Additional costs that parents have to bear for the vaccine.
    - Multiple other vaccinations pending thus rendering HPV vaccine as “less important”.
    - Inadequate time during consultation to discuss about anticipatory advice about HPV and the vaccine.
    - Lack of patient information sheets and supplementary materials for parents to refer to.
    - I feel that HPV vaccine should be administered at an older age.
    - Parental or physical concerns over side-effects of the vaccine.
    - I am hesitant to discuss about HPV infection due to its association with sexuality/sexual activity.
    - The patients under my care are unlikely to participate in sexual activity.
    - I feel that HPV vaccine should be advocated for by gynaecologists or family physicians instead.
    - Resistance from parents to discuss about HPV vaccine/parental perception of HPV vaccination being unimportant
    - The need to schedule additional return visits to complete HPV vaccine series.
    - Logistical issues in the clinics or wards in administering HPV vaccine.
11. Please rank your top 3 reasons among the above options in Q10. (Most important reason)
12. Please rank your top 3 reasons among the above options in Q10. (2nd most important reason)
13. Please rank your top 3 reasons among the above options in Q10. (3rd most important reason)
14. Which of the following options would be most effective to encourage and increase the uptake of HPV vaccine? (Tick the top 3 options)
    - Using broadcast media and/or social media (Facebook, Instagram, Youtube, playing education videos in clinics and wards)
    - Ready-to-use key messages and educational materials on HPV and vaccine for parents & patients e.g.patient information leaflets
    - Vaccine appointment reminders for parents and/or healthcare providers
    - Making HPV vaccine free
    - Holding talks about HPV and vaccine for parents
    - Dissociating HPV vaccination from precocious sexual activity in teens
    - Educating healthcare providers about HPV and vaccine, and teaching communication strategies to advocate for the vaccine
    - Others:
